# Cooperation between Monocyte-Derived Cells and Lymphoid Cells in the Acute Response to a Bacterial Lung Pathogen

**DOI:** 10.1371/journal.ppat.1005691

**Published:** 2016-06-14

**Authors:** Andrew S. Brown, Chao Yang, Ka Yee Fung, Annabell Bachem, Dorothée Bourges, Sammy Bedoui, Elizabeth L. Hartland, Ian R. van Driel

**Affiliations:** 1 Department of Biochemistry and Molecular Biology, Bio21 Molecular Science and Biotechnology Institute, University of Melbourne, Melbourne, Victoria, Australia; 2 Department of Microbiology and Immunology, University of Melbourne at the Peter Doherty Institute for Infection and Immunity, Melbourne, Victoria, Australia; Yale University School of Medicine, UNITED STATES

## Abstract

*Legionella pneumophila* is the causative agent of Legionnaires’ disease, a potentially fatal lung infection. Alveolar macrophages support intracellular replication of *L*. *pneumophila*, however the contributions of other immune cell types to bacterial killing during infection are unclear. Here, we used recently described methods to characterise the major inflammatory cells in lung after acute respiratory infection of mice with *L*. *pneumophila*. We observed that the numbers of alveolar macrophages rapidly decreased after infection coincident with a rapid infiltration of the lung by monocyte-derived cells (MC), which, together with neutrophils, became the dominant inflammatory cells associated with the bacteria. Using mice in which the ability of MC to infiltrate tissues is impaired it was found that MC were required for bacterial clearance and were the major source of IL12. IL12 was needed to induce IFNγ production by lymphoid cells including NK cells, memory T cells, NKT cells and γδ T cells. Memory T cells that produced IFNγ appeared to be circulating effector/memory T cells that infiltrated the lung after infection. IFNγ production by memory T cells was stimulated in an antigen-independent fashion and could effectively clear bacteria from the lung indicating that memory T cells are an important contributor to innate bacterial defence. We also determined that a major function of IFNγ was to stimulate bactericidal activity of MC. On the other hand, neutrophils did not require IFNγ to kill bacteria and alveolar macrophages remained poorly bactericidal even in the presence of IFNγ. This work has revealed a cooperative innate immune circuit between lymphoid cells and MC that combats acute *L*. *pneumophila* infection and defines a specific role for IFNγ in anti-bacterial immunity.

## Author Summary

Legionnaires’ Disease, a leading cause of community-acquired pneumonia resulting in significant morbidity and death, develops after infection with *Legionella* bacteria that replicate inside specialised sentinel cells of the lung. Although some factors that help combat *Legionella* infection are known, an overall view of the early immune events that are triggered by infection were unclear and we have addressed this issue here using recently developed methods. Our study implicates a number of new cells in the defence against *Legionella* infection and identifies key molecules that participate in a feedback circuit required for eradication of bacteria. In particular, we find that specific immune cells derived from blood monocytes invade the infected lung and trigger other blood-derived cells to produce the potent inflammatory mediator IFNγ. In turn IFNγ stimulates monocyte-derived cells to destroy bacteria. Surprisingly, IFNγ did not influence the behaviour of other abundant immune cells. The reported mechanism provides a basis for future investigation into the host response to combat intracellular bacteria, particularly in lung, and for assessing the risk to individuals infected with lung pathogens.

## Introduction

Innate immune responses in infected peripheral tissues are essential for controlling invading pathogens in the early phases of infection to prevent rapid pathogen replication and widespread dissemination. Despite this vital role, the main cells and factors that control innate immune responses in tissues are poorly defined. In particular, the innate functions of dendritic cells (DC) in peripheral tissues are not well understood compared to their role as antigen presenting cells in lymphoid organs and the significance of tissue-borne lymphoid cells in peripheral innate immunity has been recognized only recently. Components of the innate immune response to pathogens have mostly been studied in isolation and there are few examples where the interplay between distinct innate components that mediate pathogen clearance *in vivo* is well understood.

Ly6C^hi^ or “classical” monocytes are circulating mononuclear cells that rapidly enter inflamed tissues upon insult or infection. Here, the cells can mediate effector function whilst maintaining an undifferentiated phenotype [1], or undergo terminal differentiation upon which a proportion lose expression of Ly6C [2]. Monocyte derivatives can contribute functions that are otherwise associated with either macrophages or DC [3–5], which has led to monocyte-derived cells being referred to as monocyte-derived DC [2,5,6] or inflammatory monocytes/macrophages [7,8]. Since the exact developmental origins and functions of differentiated monocytes in inflammatory sites is usually unclear a recent proposal suggests the term monocyte-derived cell (MC) [5], which we have adopted here.

To gain an integrated understanding of the *in vivo* innate immune network in lung tissue, here we investigated the acute response to respiratory infection with the intracellular bacterial pathogen *Legionella pneumophila*. *L*. *pneumophila* is an opportunistic human pathogen and the causative agent of Legionnaires’ Disease, an acute form of pneumonia associated with high rates of morbidity and mortality [9]. Following inhalation into the lung, *L*. *pneumophila* replicates in alveolar macrophages within an intracellular vacuole that evades fusion with the endocytic pathway [10,11]. Host resistance to *L*. *pneumophila* in mice requires a rapid inflammatory response in tissue that combats bacterial replication and is stimulated by innate pattern-recognition receptors [12–14]. This is followed by an adaptive immune response mediated by T and B cells that begins ~5 days after infection [12,13,15]. The innate response to *L*. *pneumophila* is greatly compromised in the absence of effector cytokines such as IFNγ [16,17], although the cellular targets of IFNγ have not been defined.

Many studies have focussed on the role of macrophages in early immune responses to *L*. *pneumophila*, while the function of conventional DC (cDC) and MC have not been studied in detail. While facets of the cellular immune response have been investigated previously [12,13,15,16,18–22], only recently has an analysis of the temporal kinetics of lung phagocytes and lymphocytes present in the acute stages of *L*. *pneumophila* infection been made [20]. Here we applied the methodology of Lambrecht and colleagues [2] and specifically separated neutrophils, AM, cDC and the monocytic compartment, and found that neutrophils and MC were the major phagocytic cell types that interact with *L*. *pneumophila* early in infection. While MC are widely recognised as infiltrating inflamed tissue, the significance of their role during lung infection is only partially understood in part due to the difficulty of delineating MC from other DC types [2,23]. We observed that MC were recruited rapidly during lung infection. MC were required for optimal bacterial clearance by instructing lymphoid cells to produce the key cytokine IFNγ, which in turn activated the bactericidal activity of MC. This work demonstrates that MC play a key immunoregulatory and protective role during pulmonary bacterial infection and also helps to define a specific role for IFNγ.

## Results

### MC and neutrophils are the dominant cell types in the innate response to *L*. *pneumophila*

**Fig 1:**
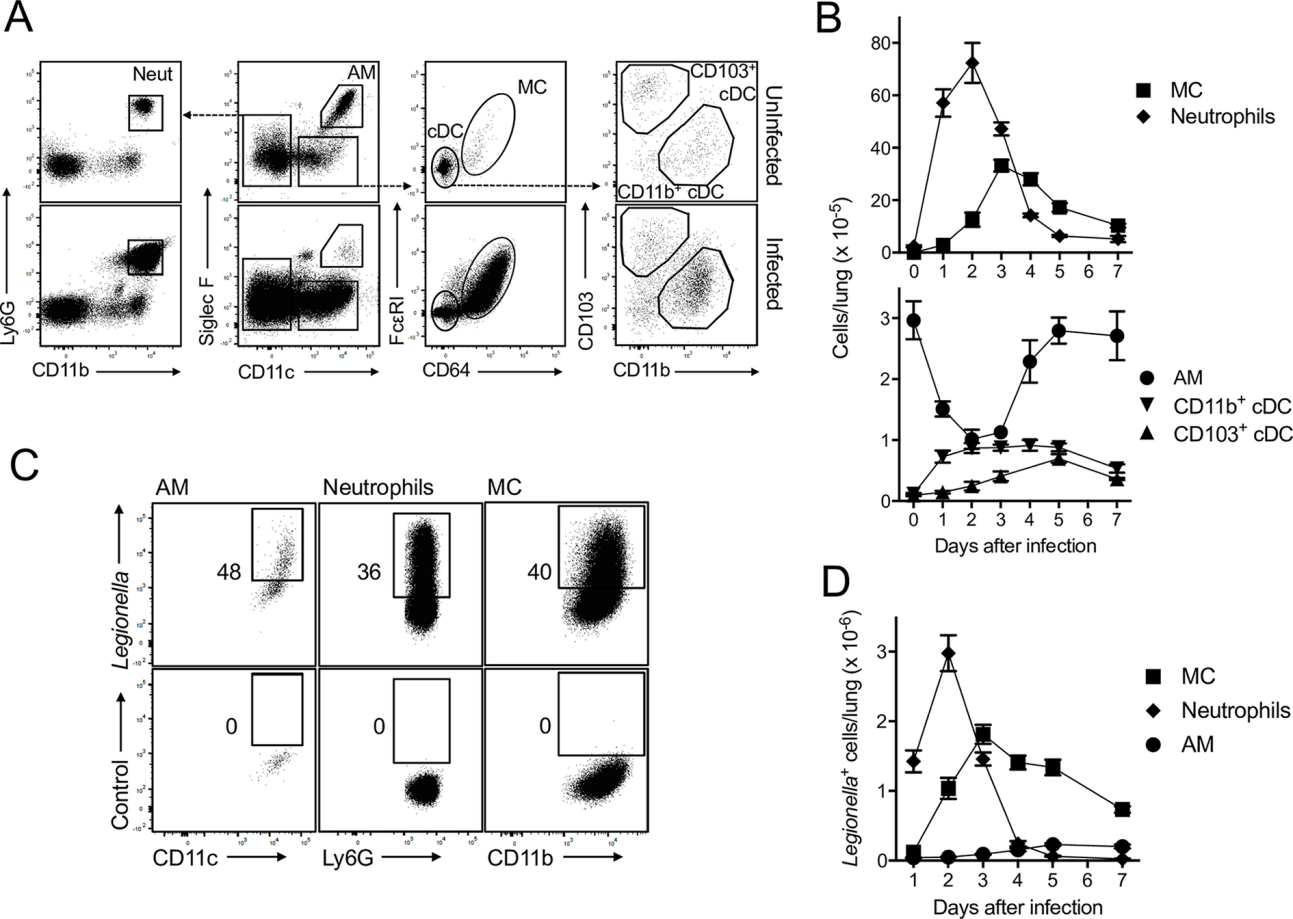
Neutrophils and MC are the dominant inflammatory phagocytic cells in lung following *L*. *pneumophila* infection. C57BL/6 mice were infected with *L*. *pneumophila* and CD45^+^ cells were analysed on day 2 after infection. A. Gating strategy to identify neutrophils (Neut), AM, MC and cDC. Upper panels, uninfected mice, Lower panels, infected mice. Dashed lines indicate the gated population is further analysed in adjacent panel. Expression of CD11b and Ly6G on CD11c^-^ cells allowed identification of neutrophils. AM, like DC are CD11c^+^, but also expressed Siglec F. Siglec F^-^CD11c^+^ cells comprised MC and cDCs and these were separated by using Fcε receptor I and CD64 as well as CD11b and CD103, respectively. B. Enumeration of the number of cells per lung for the indicated cell types. C. Cells from *L*. *pneumophila*-infected mice were stained with a *L*. *pneumophila*-specific antibody (upper panels). Lower panels, isotype control. Numbers represent percentage of cells in the gate shown. Cells were identified using the strategy shown in ‘A’. D. Enumeration of cells per lung that stained with a *L*. *pneumophila* antibody. In B and D mean ± SEM is shown. B, n ≥ 14 for all groups and pooled from ≥ 4 separate experiments. D, n ≥ 11 for all groups and pooled from ≥ 4 separate experiments.

Recent work [2,23] showing that expression of Fcε receptor I and CD64 can be used to differentiate MC and cDC has allowed an accurate appraisal of the monocytic cells that invade the lung. We used these approaches to characterise the time course of phagocyte recruitment during the early immune response to *L*. *pneumophila*. Using a gating strategy to identify neutrophils, alveolar macrophages (AM) and DC types in lung (Fig 1A), the total numbers of phagocytic cells per lung before and after infection were determined (Fig 1B). As expected, lung AM represented the major monocytic/phagocytic cell type in steady state. Both CD11b^+^ and CD103^+^ cDC were detected but very few MC were present. However, within 24 h of infection with *L*. *pneumophila*, neutrophils and MC became the dominant phagocytic cell types in the lung and by day 3 the numbers of MC were comparable to neutrophils (Fig 1B). In contrast, the number of neutrophils rapidly waned after day 2. Interestingly, the number of AM significantly decreased over the first 3 days of infection but then began to rebound at day 4 as the bacterial load lessened. (Note that bacterial number was not directly ascertained in these experiments. However in similar experiments we found that bacterial number peaks at day 1–2 and we have previously published that the number of *Legionella* ^+^ neutrophils, which peaks at day 2, is a reliable indication of bacterial load [24].) cDC increased in number and rivalled AM in abundance by day 3 (Fig 1B). Thus, contrasting with the steady state levels, MC, neutrophils and cDC outnumbered AM in the lung during acute *L*. *pneumophila* infection.

Using an anti-*L*. *pneumophila* antibody [24], we observed that large proportions of AM, neutrophils and MC had phagocytosed bacteria or material derived from the bacteria (Fig 1C shows flow cytometric plots at 2 days after infection, Fig 1D shows enumeration of the number of antibody stained cells per lung). At 24 h the vast majority of *L*. *pneumophila* staining occurred in neutrophils. However, by day 2 MC staining for *L*. *pneumophila* was comparable to neutrophils and remained at high levels through days 3–7. The number of *L*. *pneumophila*-containing MC and neutrophils was >10-fold higher than AM at days 2 and 3. cDC also stained with the anti-*L*. *pneumophila* antibody although they represented less than 1% of total *L*. *pneumophila*-positive cells at all time points.

This comprehensive analysis of phagocyte recruitment showed that neutrophils and MC were the dominant phagocytic cells present after *L*. *pneumophila* infection and were associated with bacterial material. In contrast, AM numbers rapidly decreased in the first 3 days of infection and became a minor *L*. *pneumophila-*associated cell type.

### MC were required for efficient clearance of *L*. *pneumophila* and produced IL12 that is critical for IFNγ production

**Fig 2:**
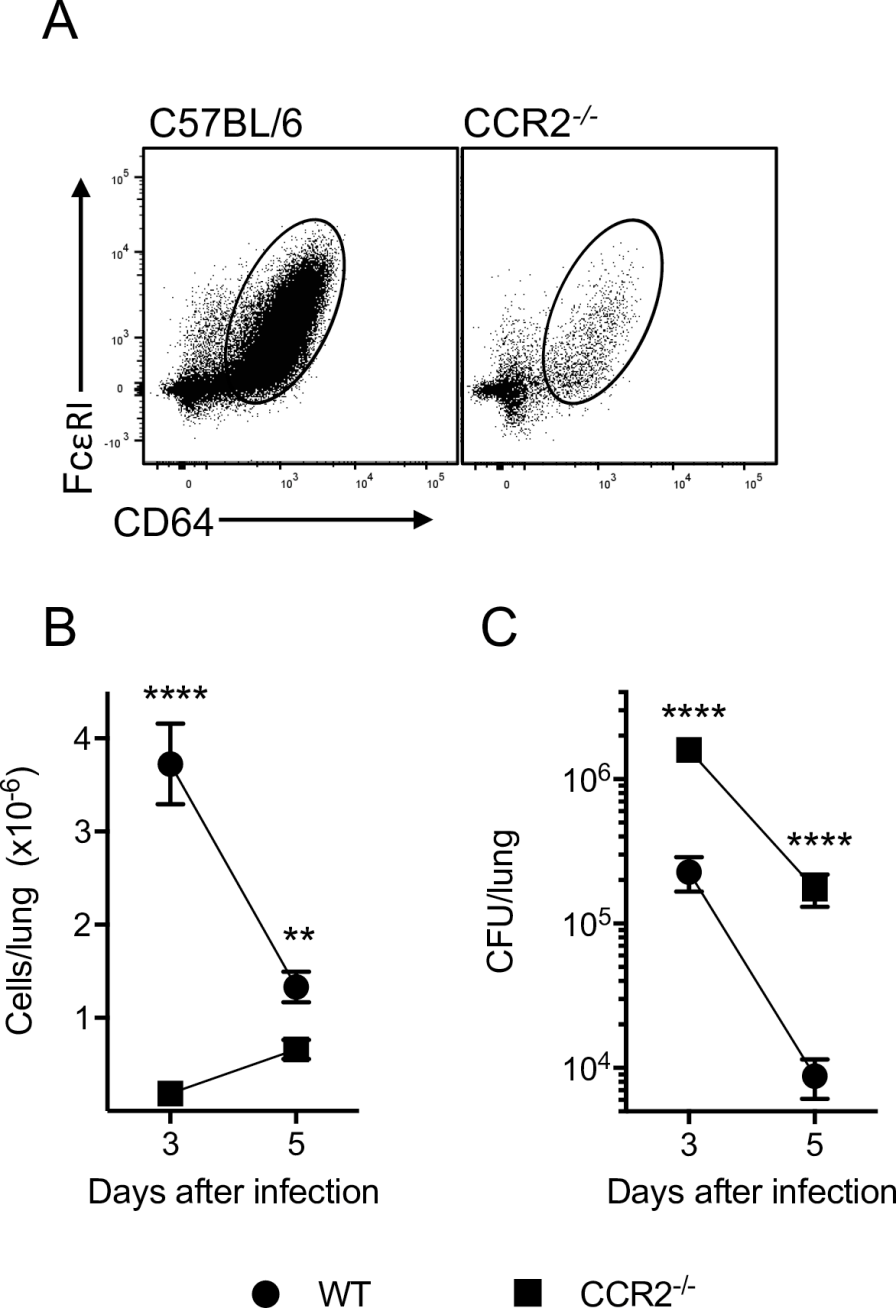
MC are required for optimal clearance of *L*. *pneumophila*. Wild type C57BL/6 or CCR2^-/-^ mice were infected with *L*. *pneumophila* and analysed for MC number in the lung (at day 2 in A and at day 3 and 5 in B) and *L*. *pneumophila* CFU in lung (C). In B and C mean ± SEM is shown. B, n ≥ 9 for all groups and pooled from ≥ 4 separate experiments. C, n ≥ 9 for all groups and pooled from ≥ 4 separate experiments. **. P < 0.01, ****. P < 0.001.

To gauge the functional importance of MC in combatting *L*. *pneumophila* infection, mice deficient in the C-C chemokine receptor 2 (CCR2^-/-^ mice [6,25]) were infected and bacterial load was assessed (Fig 2). Monocytes in CCR2^-/-^ mice have an impaired ability to exit the bone marrow and, hence, to infiltrate tissue and convert to MC [25]. We observed that MC were significantly reduced in the lungs of CCR2^-/-^ mice infected with *L*. *pneumophila* at days 3 and 5 (Fig 2A and 2B), and this was associated with a ~10-20-fold increase in bacterial burden 3 and 5 days after infection (Fig 2C **)**. Of the other inflammatory cell types, neutrophils were significantly increased on day 3 and 5 in CCR2^-/-^ mice, which would not be expected to contribute to an increase in bacteria, and CD11b^+^ cDC showed a small decrease at day 3. No other significant differences were found (S1 Fig).

**Fig 3:**
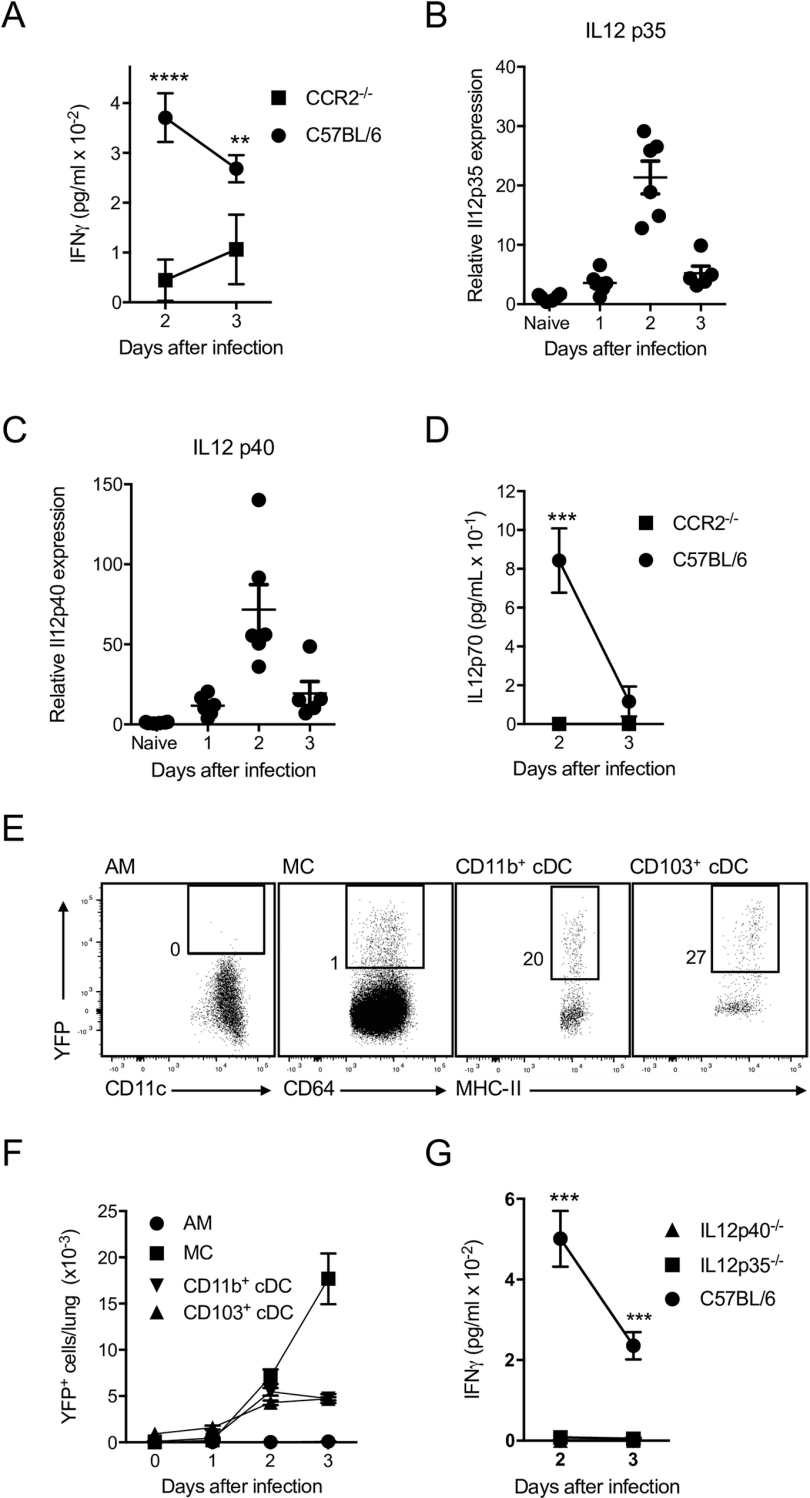
MC are a major source of IL12, a key driver of IFNγ production in lung after *L*. *pneumophila* infection. C57BL/6 mice (B,C), IL12p40-YFP mice (E,F) or the indicated mouse strains (A,D,G) were infected with *L*. *pneumophila*. A, D, G. IFNγ (A, G) or IL12p70 (D) levels were measured in BALF of the indicated strains. B, C. Quantitative PCR was used to detect transcripts in whole lung samples for IL12p35 and IL12p40 as shown. Each dot represents the reading for one mouse. E, F. IL12p40-YFP reporter mice were used to analyse expression of IL12p40. Representative flow cytometric plots for the cell types indicated are shown in E (Numbers represent percentage of cells in the gate shown. Gates were set by analysis of infected wild type mice) and enumeration of cells is shown in F. In A, D, F, G mean ± SEM is shown. A. n ≥ 9 for all groups and pooled from 4 separate experiments. B, C. Data pooled from 2 separate experiments. D. n ≥ 9 for all groups and pooled from 4 separate experiments. F. n ≥ 6 for all groups and pooled from 4 separate experiments. G. n ≥ 7 for all groups and pooled from 4 separate experiments. *, P < 0.05, **. P < 0.01, ***. P < 0.005. ****. P < 0.001.

To understand the impact of reduced MC numbers during infection with *L*. *pneumophila*, we analysed cytokine profiles in the bronchoalveolar lavage fluid (BALF) of CCR2^-/-^ mice. The levels of IFNγ, a cytokine known to be important for resistance to *L*. *pneumophila* infection [16,17] were greatly reduced in the BALF of CCR2^-/-^ mice (Fig 3A). To resolve the factors that drive the secretion of IFNγ, we focussed on IL12, which is a known inducer of IFNγ [26]We found that expression of mRNAs for the two subunits of IL12p70 [27] IL12p35 and IL12p40, were induced in the lung 24 h after *L*. *pneumophila* infection and peaked at day 2 (Fig 3B and 3C). In CCR2^-/-^ mice, we found that IL12p70 was not detectable in the BALF upon *L*. *pneumophila* infection (Fig 3D), suggesting that MC were a major source of IL12p70. To further characterise IL12 expression, we infected IL12p40-enhanced yellow fluorescent protein (IL12p40-YFP) reporter mice [28] with *L*. *pneumophila*. By staining *in vitro* stimulated DC with anti-IL12p40 antibodies, we confirmed that these mice faithfully report IL12p40 expression (S2A Fig). Although CD103^+^ cDC, CD11b^+^ cDC and MC produced some IL12p40 (Fig 3E and 3F), MC predominated in the response (Fig 3F), supporting the conclusion that MC were a major source of IL12. Surprisingly, AM did not produce detectable IL12p40 (Fig 3E and 3F). Consistent with IL12 playing a major role in the induction of IFNγ, IL12p35-deficient mice (IL12p35^-/-^) and IL12p40-deficient mice (IL12p40^-/-^) infected with *L*. *pneumophila* had greatly reduced levels of IFNγ in the BALF (Fig 3G). Hence, MC play a major role in clearance of *L*. *pneumophila* and are an important source of IL12, which is largely responsible for inducing IFNγ after infection (Figs 2 and 3).

As IL18 also induces IFNγ secretion, we assessed the role of IL18 in the response to *L*. *pneumophila*. IL18^-/-^ mice contained reduced levels of IFNγ in BALF compared to C57BL/6 mice (S3A Fig). However, bacterial clearance in IL18^-/-^ mice was not significantly altered (S3B Fig) suggesting that although reduced, the level of IFNγ produced in the absence of IL18 was still sufficient to stimulate optimal clearance of bacteria. These data are in agreement with previous findings [16,21,29].

### IFNγ is required for the bactericidal activity of MC but not neutrophils

**Fig 4:**
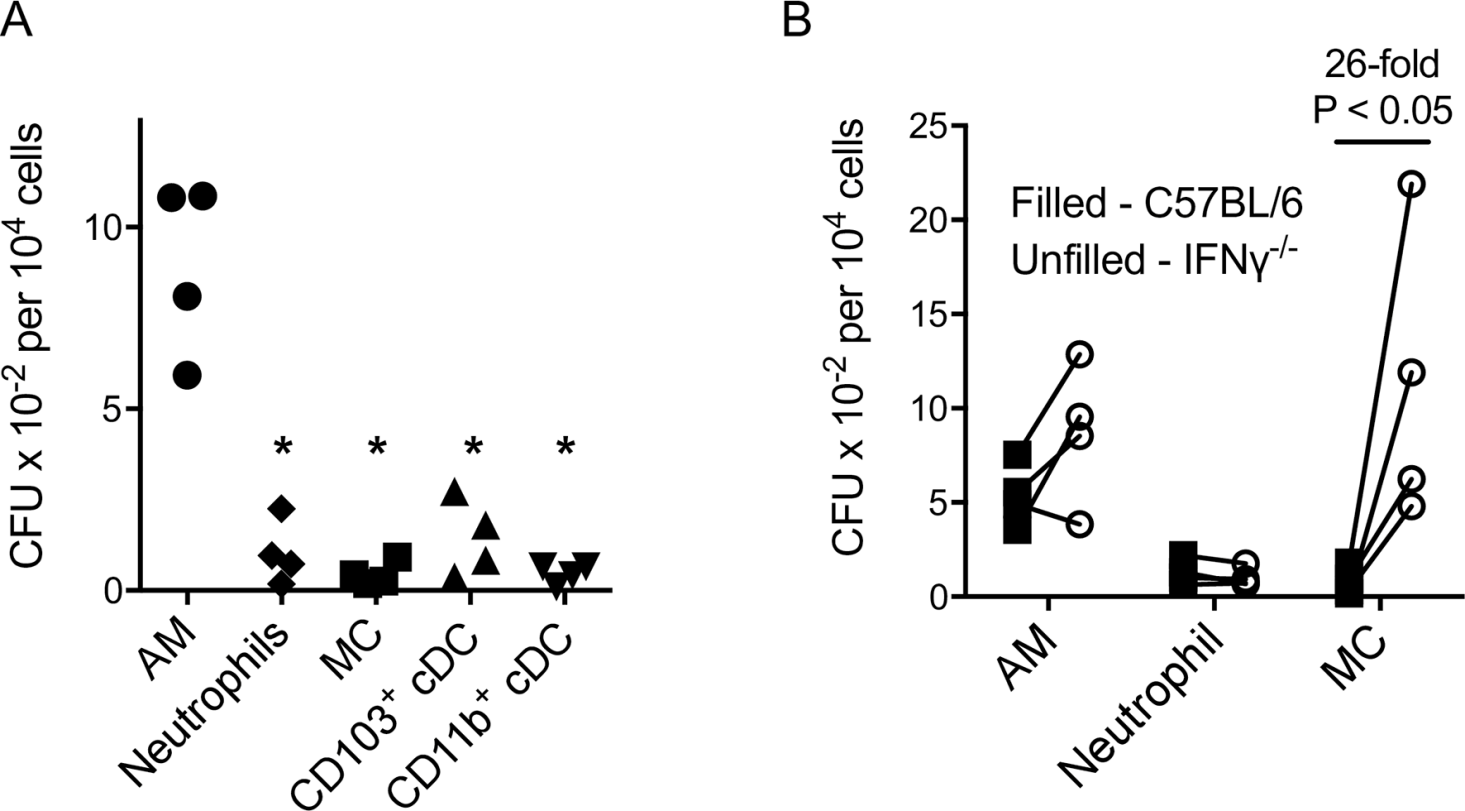
Optimal bactericidal activity of MC, but not neutrophils, is dependent upon IFNγ. C57BL/6 (A, B) or IFNγ^-/-^ (B) mice were infected with *L*. *pneumophila* and at 2 days after infection the indicated cell types were isolated, lysed and the lysates cultured on selective bacterial culture plates to determine the number of *L*. *pneumophila* CFU per 10^4^ cells in each cell population. In B, lines connect data points from individual experiments using the same bacterial inoculum. Each point represents the results from one experiment where lungs from 4–5 mice were pooled. *. P < 0.05. In A all comparisons to AM group. In B, the fold change refers to the ratio of the averages of the two groups.

To determine which phagocyte types support *L*. *pneumophila* replication *in vivo* and gauge their bactericidal activity, phagocytes were purified from the lungs of wild type mice 2 days after infection, lysed and plated on bacteriological plates for enumeration of *L*. *pneumophila* colonies (CFU) (Fig 4A). The number of viable bacteria recovered on a per cell basis was relatively high for AM but low in neutrophils, MC and cDCs, suggesting that AM are poorly bactericidal and constitute the major site for replication of *L*. *pneumophila*, which consistent with previous work [20]. We also examined if bacterial survival in AM, neutrophils and MC was influenced by IFNγ by repeating the above experiment with cells isolated from infected lungs of wild type and IFNγ^-/-^ mice (Fig 4B). While IFNγ deficiency made no difference to the number of viable bacteria isolated per cell from AM or neutrophils, 26-fold more viable bacteria were recovered from MC from IFNγ^-/-^ mice compared to wild type mice (Fig 4B). Note that these differences were not due to alterations in bacterial load in lung tissue, because the number of viable bacteria in lungs of C57BL/6 mice and IFNγ^-/-^ mice at 2 days after infection was not significantly different (C57BL/6, 1.22 x 10^6^ ± 3.45 x 10^5^; IFNγ^-/-^, 1.39 x 10^6^ ± 2.6 x 10^5^; n = 5, NS).

These data suggest that AM are efficient replicative hosts of *L*. *pneumophila in vivo* even in the presence of IFNγ, a finding that differs from *in vitro* studies in which macrophage cell lines efficiently kill *L*. *pneumophila* following activation by IFNγ [30] The anti-bacterial activity of neutrophils does not require IFNγ. In contrast to both AM and neutrophils, optimal control of bacterial numbers by MC required IFNγ stimulation thus defining a significant cellular target for this cytokine. These data also suggest that MC contributed to *L*. *pneumophila* clearance both through their direct IFNγ-dependent bactericidal activity and their ability to stimulate IFNγ production via IL12.

### Lymphoid cells are the source of IFNγ immediately after *L*. *pneumophila* infection

**Fig 5:**
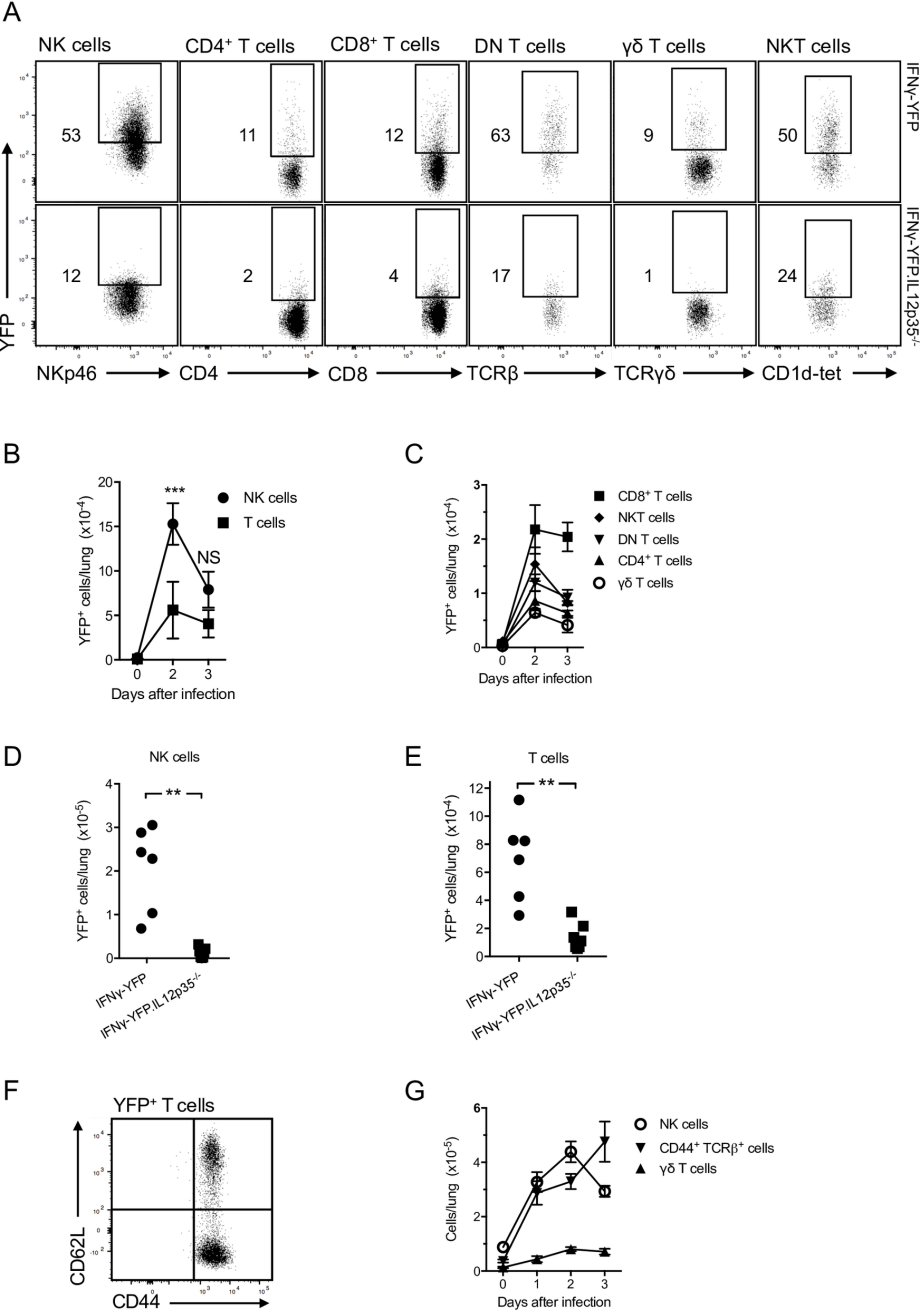
NK cells and memory T cells produce IFNγ in the acute response *L*. *pneumophila*. IFNγ-YFP reporter mice (GREAT mice, A-F), IFNγ-YFP.IL12p35^-/-^ (A,D,E) or C57BL/6 mice (G) were infected with *L*. *pneumophila* and analysed on day 2 (A, D-F) or as indicated (B, C, G). A. YFP fluorescence in the cell types indicated. NK cells were defined as NK1.1^+^NKp46^+^CD3^-^, T cells as CD3^+^TCRβ^+^ or CD3^+^TCRγδ^+^ and NKT cells as TCRβ^+^ cells that stained with a CD1d-tetramer. Indicated gates set by analysis of wildtype mice. B-E. Enumeration of NK and T cells from IFNγ-YFP reporter mice (B, C) or the mouse strains indicated (D, E). F. CD62L and CD44 expression of all IFNγ-YFP^+^CD3^+^TCRβ^+^ cells. G. Enumeration of NK cells, CD44^+^CD3^+^TCRβ^+^ T cells (CD44^+^TCRβ^+^ cells, which includes conventional T and NKT cells) and γδ T cells. In A and F the numbers represent percentage of cells in the gate shown. In B, C, G. mean ± SEM is shown. B, n ≥ 4 for all groups and pooled from 4 separate experiments. C, n ≥ 4 for all groups and pooled from 5 separate experiments. In D, E, each dot refers to one mouse. D, n ≥ 6 for all groups and pooled from 2 separate experiments. E, n ≥ 6 for all groups and pooled from 2 separate experiments. G, n ≥ 7 for all groups and pooled from ≥ 3 separate experiments **. P < 0.01, ***. P < 0.005.

To identify the source of IFNγ during *L*. *pneumophila* infection, IFNγ-YFP reporter mice (GREAT mice [31]) were used to detect IFNγ-producing cells in lung. We verified that these mice faithfully report IFNγ production using antibody staining (S2B Fig). At steady state, YFP expression was not detected in any cell type (Fig 5). In mice infected with *L*. *pneumophila* 2 days earlier, IFNγ-YFP expression was found in NK cells and T cells and not any other cell type in lung. While there have been previous reports of individual sub-types of T cells contributing to the innate response [32–39], we examined the totality of the innate T cell response to *L*. *pneumophila*. For T cells, CD8^+^, NKT, CD4^+^, CD4^-^CD8^-^ (DN) and γδ cells all contributed to IFNγ production (Fig 5A, upper panels). The proportions of cells that produced IFNγ varied between cell types with ~30–80% of NKT and DN T cells producing IFNγ while ~ 5–15% of CD8^+^, CD4^+^ and γδ T cells were IFNγ-YFP^+^. Less than 10% of DN T cells stained with an MR1-tetramer and thus were mucosal-associated invariant T cells, however less than 4% of these MR1-tetramer stained cells expressed IFNγ-YFP. Quantitation of the number of IFNγ producing cells is shown in Fig 5B and 5C. At 2 days after infection, IFNγ^+^ NK cells predominated but by day 3 the number of IFNγ producing NK and T cells were similar. CD8^+^ T cells appeared to be the predominant T cell subset producing IFNγ particularly at day 3 (Fig 5C). Consistent with the findings discussed above, this IFNγ production depended on IL12, as demonstrated by crossing the IFNγ-YFP mice onto an IL12p35^-/-^ background (IFNγ-YFP.IL12p35^-/-^, Fig 5A, 5D and 5E). For all cell types IFNγ production was largely IL12-dependent, in agreement with results from IL12^-/-^ and CCR2^-/-^ mice (Figs 2 and 3).

We next examined if IFNγ-producing T cells found in the lung were naïve or had previously been activated by antigen. Almost all T cells that were IFNγ^+^ also expressed CD44 (Fig 5F), suggesting they were antigen-experienced and most likely memory T cells. (Note that NKT cells and γδ T cells constitutively express CD44). It seems likely that these CD44^+^ T cells were infiltrating circulating T effector/memory cells rather than T resident memory (Trm) cells as less than 3% of the IFNγ-secreting T cells expressed the typical Trm markers CD103 and CD69 [40]. The conclusion that the memory T cells infiltrated the lung subsequent to infection rather than being tissue resident was supported by analysis of the number CD44^+^ T cells in tissue over time. CD44^+^TCRβ^+^ T cells were found at very low levels in steady-state lung but after infection infiltrated the tissue (Fig 5G). The same was true for NK cells and γδ T cells.

### IFNγ production by T cells does not require TCR stimulation and can limit *L*. *pneumophila* infection in the acute response

**Fig 6:**
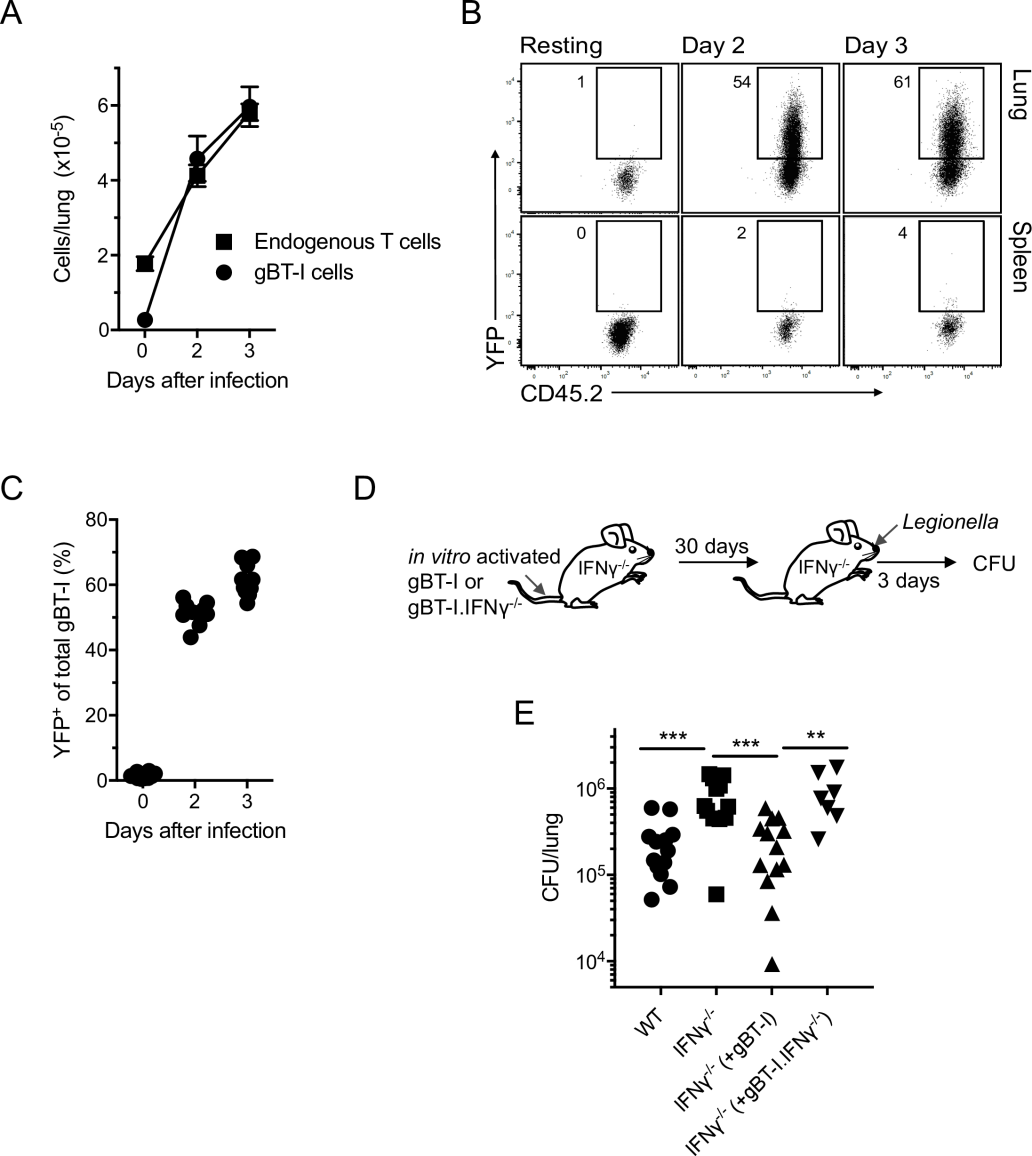
IFNγ production by memory T cells in the acute phase of the response to *L*. *pneumophila* is non-cognate and can contribute to bacterial clearance. A-C. C57BL/6 mice were injected with 10^7^ gBT-I.IFNγ-YFP T cells that had been stimulated *in vitro* with antigen. After 30 days mice were infected with *L*. *pneumophila*. A. Number of T cells derived from the recipient mice (Endogenous) and gBT-I T cells in lungs at the times shown. Cells were identified using congenic markers. Mean ± SEM is shown. n ≥ 10 for all groups and pooled from 4 separate experiments. B. Flow cytometric profiles of YFP expression of gBT-I.IFNγ-YFP T cells in lung and spleen at the days shown. Indicated gates set by analysis of endogenous cells. C. Proportion of gBT-I.IFNγ-YFP T cells in lung expressing YFP on the days shown. D. Experimental design to examine the effectiveness of IFNγ produced by memory T cells in *L*. *pneumophila* clearance. See text for further details. E. Results of experiment shown in ‘D’. WT, wild type C57BL/6 mice without transfer of T cells. IFNγ^-/-^, IFNγ^-/-^ mice without transfer of T cells. IFNγ^-/-^ (+gBT-I), IFNγ^-/-^ mice that received IFNγ-sufficient gBT-I T cells. IFNγ^-/-^ (+gBT-I.IFNγ^-/-^), IFNγ^-/-^ mice that received T cells from gBT-I.IFNγ^-/-^ mice. CFU/lung at day 3 after infection are shown. In C and E, each symbol represents data for one mouse. C, Data pooled from 4 separate experiments. D, Data pooled from 4 separate experiments. **. P < 0.01, ***. P < 0.005.

The high proportion of T cells expressing IFNγ and the rapidity of this response led us to suspect that T cells were being stimulated independently of TCR engagement (non-cognate stimulation). To determine if expression of IFNγ required TCR stimulation, CD8^+^ T cells from a mouse strain expressing a TCR specific for herpes simplex virus antigen gB and also containing the IFNγ-YFP reporter (gBT-I.IFNγ-YFP mice) were activated *in vitro* and seeded into wild type mice and allowed to convert to memory T cells over 30 days. Approximately 90% of these cells initially produced IFNγ 4 days after activation with antigen *in vitro*, but after ~20 days in the wild type host, expression of the IFNγ reporter gene was undetectable and ~99% expressed CD44 indicating a memory T cell phenotype. At 30 days after transfer the mice were infected with *L*. *pneumophila*. The number of transferred gBT-I.IFNγ-YFP T cells that infiltrated the lungs of mice was comparable to that of endogenous T cells at 2–3 days after *L*. *pneumophila* infection (Fig 6A) and ~ 50–60% of these cells expressed YFP (Fig 6B and 6C) indicating that cognate MHC-peptide stimulation of the TCR was not required for activation of the memory T cells in the lung to drive IFNγ secretion. Also of note, very few IFNγ-YFP^+^ cells were detectable in spleen indicating that stimulation was localized to the lung and not a systemic phenomenon (Fig 6B).

To determine if non-cognate production of IFNγ could contribute to pathogen clearance, T cells from gBT-I mice or gBT-I mice on a IFNγ^-/-^ background (gBT-I.IFNγ^-/-^ mice) were activated with gB_498-505_ *in vitro*, transferred into IFNγ^-/-^ mice and allowed to develop into memory T cells over 30 days (Fig 6D). Accordingly, the wild type gBT-I T cells were the only cells capable of producing IFNγ in these mice. IFNγ^-/-^ mice had a significantly higher bacterial load than wild type mice (Fig 6E). Three days after *L*. *pneumophila* infection, mice that had received IFNγ-sufficient gBT-I T cells had a bacterial load comparable to wild type mice while mice that received the gBT-I.IFNγ^-/-^ T cells had a bacterial burden similar to that of IFNγ^-/-^ mice without T cell transfer (Fig 6E). These data showed that non-cognate stimulation of T cells was sufficient to control bacterial load in the lung and that the production of IFNγ was required for this action.

## Discussion

The lung is a major site for potential infection by pathogens. It is therefore important to gain an understanding of the inflammatory milieu in the lung following infection. In this work we examined the cellular interplay following lung infection with *L*. *pneumophila*. *L*. *pneumophila* is the causative agent of Legionnaires’ disease, a potentially fatal pneumonia that results from environmental exposure to the bacteria. Upon entering the lung, *L*. *pneumophila* replicates inside alveolar macrophages. Intracellular replication requires the bacterial Dot/Icm type IV secretion system to inject bacterial effector proteins into the macrophage cytosol, which establishes a ‘*Legionella* containing vacuole’ permissive for bacterial replication [10,11,41].

Until recently, the inability to reliably identify inflammatory cell types, particularly myeloid cells, in lung has made it difficult to analyse cellular responses during infection. However, newly identified marker sets allow accurate differentiation of lung macrophages, MC and DC subtypes [2]. Here we applied these techniques to study responses to *L*. *pneumophila* lung infection in mice and quantitated the immune cell types recruited during acute *L*. *pneumophila* infection. At 1 day after infection, and presumably hours before that time, AM represented a minor proportion of inflammatory phagocytic cells in the lung and by day 2 represented only ~ 1% of inflammatory phagocytes. To identify which phagocytes had internalised *L*. *pneumophila*, cells were stained with an anti-*L*. *pneumophila* antibody. At day 2 and 3 after infection over 97% of stained cells were neutrophils or MC. Similar to a recent study [20], we found that much higher numbers of live bacteria could be recovered from lysed AM than other phagocytic cells in the lung, on a per cell basis. This suggested that *in vivo* AM have a relatively poor ability to kill ingested *L*. *pneumophila*, even when stimulated by inflammatory cytokines found in the infected lung. IFNγ did not influence the bactericidal activity of AM as the recovery of live bacteria was equivalent from AM isolated from wild type and IFNγ^-/-^ mice. This contrasts with *in vitro* studies in which macrophage cell lines efficiently kill *L*. *pneumophila* following activation by IFNγ [30].

We also found that in contrast to other phagocytes, the number of AM rapidly decreased after infection. At the peak of infection (days 2 and 3), AM levels were ~1/3 of those found prior to infection and numbers recovered as the bacterial burden waned. AM arise from tissue-resident precursor cells seeded during embryogenesis and are not replenished by the ingress of myeloid cells [42,43], so presumably this decrease is due to an increased rate of AM death that can not be compensated by an increased rate of *in situ* generation. Regardless of the mechanism, a decrease in AM numbers during infection may contribute to control of *L*. *pneumophila* infection by limiting the replicative niches, as proposed for *Salmonella enterica*, serovar Typhimurium [44]. Nogueira *et al*. [45] suggested that *L*. *pneumophila* infection may induce rapid apoptosis of DC to limit bacterial replication. However, it is not clear to what extent such a mechanism occurs *in vivo* given the relative rarity of cDC and their poor ability to support *L*. *pneumophila* replication. Additionally, death of AM may also contribute inflammatory mediators such as IL1β and death associated molecular patterns that initiate the inflammatory response. Therefore, our work suggests that AM are probably amongst the first cell types to phagocytose *L*. *pneumophila* after lung infection and may well be pivotal in initiating the inflammatory response they appear to play a more minor role in the mass clearance of bacteria at the height of the acute infection.

MC develop in inflamed tissues from bone marrow-derived monocytes that flood into tissues in response to inflammatory signals [6,46,47]. We found that MC accumulated in the *L*. *pneumophila*-infected lung more slowly than neutrophils, but by day 3 were present in numbers comparable to neutrophils. MC have been shown to be involved in immunity to lung infections by *Klebsiella pneumoniae* [48] and *Mycobacterium tuberculosis* [8,49] as well as in the spleen after infection with *Listeria monocytogenes* [6] and *Brucella melitensis [50]*. MC were highly phagocytic and by day 3 were the dominant population associated with *L*. *pneumophila*. MC played a significant role in *L*. *pneumophila* clearance because mice with reduced infiltration of MC in lung (CCR2^-/-^) suffered significantly higher bacterial burden. One mechanism by which MC contributed to *L*. *pneumophila* clearance is by their direct bactericidal activity, which has been documented for other bacterial species [6,46]. In this work we found that optimal bacterial killing by MC required stimulation by IFNγ as lower levels of viable bacteria were recovered from MC isolated from infected wild type mice compared to infected IFNγ^-/-^ mice. In fact, numbers of viable bacteria in MC from IFNγ^-/-^ mice were comparable to that isolated from AM. This was in contrast to neutrophils where low numbers of viable bacteria were recovered from both wild type and IFNγ^-/-^ neutrophils, indicating that the bactericidal pathways used by neutrophils were not dependent upon IFNγ stimulation.

MC appear to persist in lung for a longer time after infection than neutrophils with *L*. *pneumophila* ^+^ MC present in lung beyond 7 days after infection. The continued presence of MC may be important in maintaining immunity during the transition from the innate to the critical adaptive response. Additionally, the immunoregulatory and antigen presentation activities of MC in the infected lung may play a role in the adaptive response for example by reactivating antigen specific T cells and inducing cytokine secretion [51].

MC also played an important role in stimulating early production of the effector cytokine IFNγ in response to *L*. *pneumophila*. CCR2^-/-^ mice had very low levels of IFNγ and IL12 in lung and subsequent work showed that MC were major producers of IL12 in infected lung tissue.

While it was previously shown that NK cells produce IFNγ after *L*. *pneumophila* infection [16], here we show that various T cell lineages including memory T cells as well as NKT and γδ T cells, also made significant contributions to IFNγ production. Approximately 65% of IFNγ-producing T cells were ‘conventional’ TCRαβ memory T cells, the majority of which were CD8^+^ T cells. These memory T cells made IFNγ very rapidly, and up to 80% of some T cell sub-types produced IFNγ in mice that had not previously seen *L*. *pneumophila* antigens. This led us to conclude that the T cell stimulation did not require classical TCR-MHC-peptide engagement, in other words was the result of non-cognate stimulation. To support this we found that mice seeded with memory T cells specific for an irrelevant antigen could produce a robust IFNγ response after *L*. *pneumophila* infection, thus confirming that TCR stimulation was not required for IFNγ production. Furthermore, we demonstrated that IFNγ produced by non-cognate stimulation of T cells could effectively substitute for other IFNγ sources to enable optimal control of *L*. *pneumophila* infection. Therefore, it appears that memory T cells can be considered *bona fide* members of the lymphoid armamentarium during acute responses. In other studies non-cognate production of IFNγ by CD8^+^ T cells was shown to be induced by IL12 and IL18 in combination [36], or IL18 alone [32], by CD4^+^ T cells in response to IL18 and IL33 [37,38]. In our system IFNγ production was largely IL12-dependent but we cannot rule out the possibility that IL12 acts in concert with other molecules. Indeed, we observed ~ 3-fold less IFNγ in lung after *L*. *pneumophila* infection of IL18^-/-^ mice although the clearance of the bacteria was not influenced by a lack of IL18, a result consistent with previous studies [21,29]. We did not investigate if IL18 stimulated particular lymphoid cells to secrete IFNγ.

While there have been previous reports of sub-types of T cells contributing to the innate response [32–39], this study examined the totality of the innate T cells response to *L*. *pneumophila*. One novel finding here is the contribution of DN T cells to innate immunity. While these cells were present at low total number in infected tissue, a very large proportion, ~45–80%, produced IFNγ. The origin of these DN cells is uncertain. A small proportion of the DN T cells stained with MR1 tetramer and are thus likely to be MAIT cells, but only very few of these cells expressed YFP. DN T cells have also been shown to arise from self-reactive CD8^+^ T cells [52] and can rapidly produce inflammatory cytokines [53] and our findings here may indicate a hitherto unsuspected role for these cells in protective innate immunity. Our work indicates that the sources of IFNγ are more numerous than previously thought and it is likely that there is some level of redundancy. The relative contribution of each cell type probably depends on the relative abundance in the lung after infection that would be influenced by a number of circumstances such as previous immunological experience, the environment and the microbiota.

**Fig 7:**
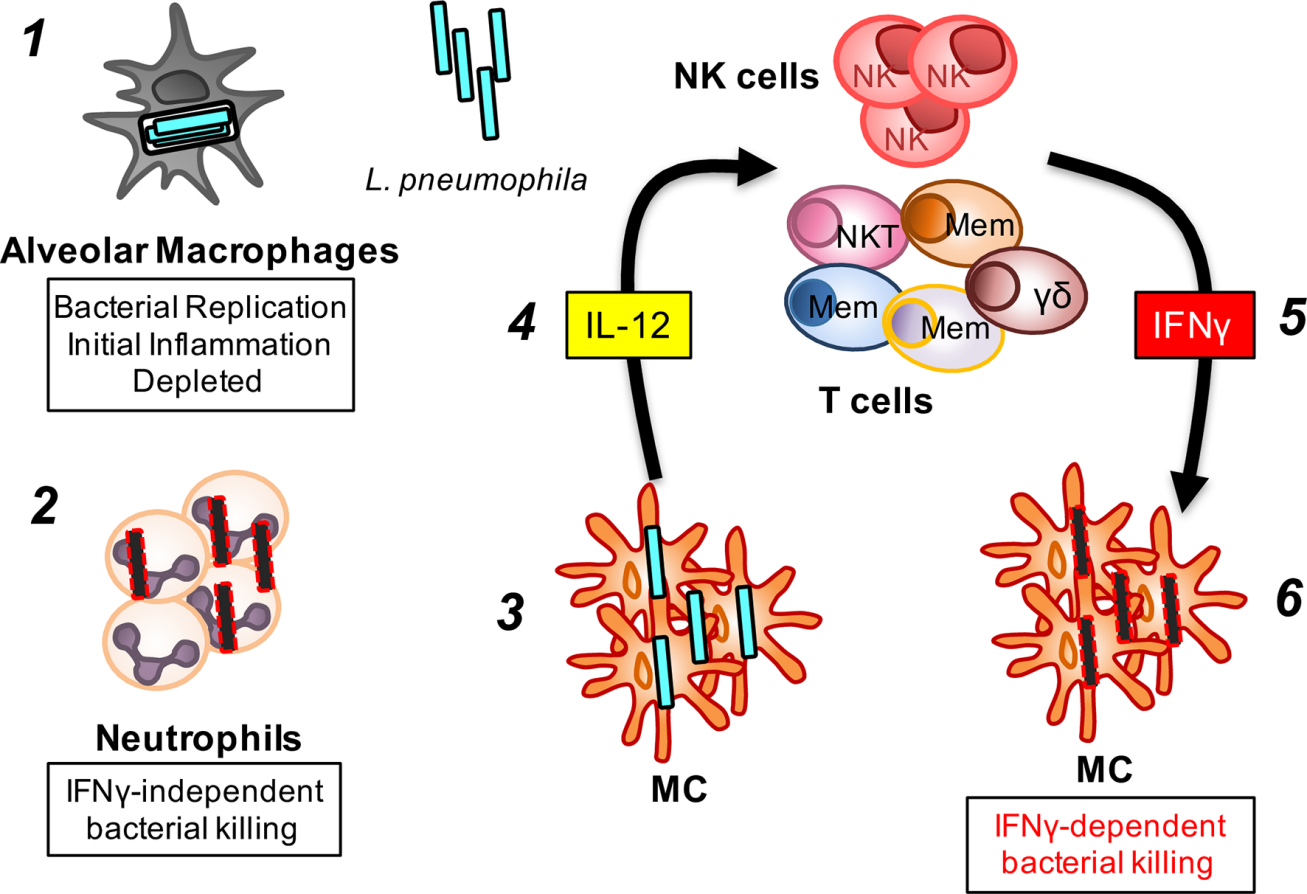
An innate immune network in the acute response to a bacterial lung pathogen. We propose the following model for the role and interactions of phagocytic and lymphoid cells in the acute phase of *L*. *pneumophila* infection. ***1*.** Tissue resident alveolar macrophages and conventional dendritic cells (not shown) are the first to engulf bacteria and produce inflammatory mediators such as cytokines and chemokines. Alveolar macrophages support replication of *L*. *pneumophila* but are also rapidly depleted, perhaps by cell death mechanisms, and may release inflammatory death-associated molecular patterns to potentiate the immune response. ***2*.** Neutrophils flood into the lung in response to the inflammatory stimuli. Neutrophils engulf and kill bacteria from early stages of infection and don’t require activation by IFNγ. ***3*.** Slightly after neutrophils, monocytes infiltrate and develop into monocyte-derived cells (MC) which then build to become the dominant phagocytic cell type. MC contribute to bacterial clearance by production of IL12 (***4***), which in turn stimulates IFNγ production by NK cells and various populations of memory T cells, NKT cells and γδ T cells. The different coloured cells labelled ‘Mem’ represent different types of memory T cells. (***5***). IFNγ is critical in stimulating bactericidal activity of MC (***6***), an activity that appears critical for optimal bacterial clearance.

Based upon these and other studies, we propose the following model for the role and interactions of phagocytic and lymphoid cells in the acute phase of *L*. *pneumophila* infection (Fig 7). Tissue resident phagocytic cells, namely, AM and conventional DC first engulf bacteria and produce inflammatory mediators such as cytokines and chemokines. AM are rapidly depleted, and may release inflammatory death-associated signals to potentiate the immune response. The decrease in AM may also act as a mechanism to limit *L*. *pneumophila* replication. Neutrophils and monocytes infiltrate the lung early in the response to inflammatory stimuli whereupon neutrophils effectively engulf and kill bacteria without requiring activation by IFNγ. Monocytes develop into mature MC *in situ* and become the dominant persistent phagocytic cell type. MC contribute to bacterial clearance by production of IL12, which in turn stimulates NK cells and various populations of memory T cells, NKT cells and γδ T cells locally to produce IFNγ. IFNγ stimulates the bactericidal activity of MC, an activity that appears critical for optimal bacterial clearance.

Overall, our findings contribute to a growing body of knowledge on the events in infected tissues that contribute to immunity to pathogenic organisms. A greater understanding of the cell types in infected tissues and their interplay may lead to an appreciation of the basis for sensitivity and resistance to pathogens and lead to more directed and effective therapies.

## Materials and Methods

### Mice

All mice were bred under specific pathogen-free conditions. C57BL/6 were used as the wild type strain and all other strains were either created on a C57BL/6 background or had been backcrossed to C57BL/6 for at least 10 generations. B6.129S4-*Ccr2* ^*tm1Ifc*^ (CCR2^-/-^) [25], B6.129-*Il12b* ^*tm1Lky*^ (IL12p40-YFP) [28], B6.129S7-*Ifng* ^*tm1Ts*^ (IFNγ^-/-^), B6.129S1-*Il12a* ^tm1Jm^ (IL12p35^*-/-*^), B6.129S1-*Il12b* ^*tm1Jm*^ (IL12p40^*-/-*^) [54], C.129S4(B6)-*Ifng* ^tm3.1Lky^ (GREAT, IFNγ-YFP) [31], Tg(TcraHsv2.3,TcrbHsv2.3)L118-1Cbn (gBT-I) [55], B6.129P2-*Il18* ^*tm1Aki*^ [56] mice were used in this study.

### L. pneumophila

*L*. *pneumophila* JR32 Δ*flaA* [19] was used for all experimental procedures in this study. For animal infection, *L*. *pneumophila* was cultured under optimal conditions on selective buffered charcoal yeast extract (BCYE) agar. Bacterial inoculum was generated by collecting colonies in PBS and adjusting via UV-spectroscopy. In all experiments mice were administered 2.5x10^6^ CFU in PBS via the intranasal route under controlled isoflurane induced anaesthesia.

To quantitate *L*. *pneumophila* in lung samples the right lobes were collected and homogenised in PBS, followed by lysis with 0.1% w/v saponin for 30 minutes at 37°C and *L*. *pneumophila* were enumerated by serially diluting the homogenate in PBS and plating onto selective BCYE.

### Flow cytometry

Lungs were prepared for flow cytometry analysis as previous [24]. Briefly, lung tissue was minced and digested by resuspension and gentle pipetting in RPMI-1640 (Gibco) with 3% v/v FCS (Gibco), 1 mg/mL DNAseI (Sigma Aldrich) and 1 mg/mL Collagenase-III (Worthington Biochemical). Undigested material was filtered with 70 μm filters (Corning) to produce single cell suspensions. Single cells were stained using antibodies and tetramers described in S1 Table. Intracellular *L*. *pneumophila* staining was as described [24]. Briefly, lung cells were fixed and permeabilised using the Fixation/Permeabilisation Kit (eBioscience) as per the manufacturers instructions, and cells stained using a polyclonal FITC-anti-*Legionella* antibody (ViroStat). Total numbers for each cell type were enumerated from the lung by addition of a known quantity of APC-labelled microspheres (BD Calibrite) to each sample prior to flow cytometry analysis.

### Bronchoalveolar lavage and cytometric bead array

BALF samples were obtained by injecting and recovering 1.5 mL chilled PBS into lungs and pelleting cells and debris. The resulting supernatant was used to analyse cytokines and chemokines via a BD cytometric bead array flex kit as per the manufacturer’s instructions.

### Quantitative reverse transcriptase polymerase chain reaction

For qRT-PCR analysis, right lung tissue was collected into RNAlater (Sigma), homogenised in TRIsure TRI-reagent (Bioline) and mRNA extracted via phase separation and precipitation using chloroform and isopronanol, respectively. mRNA (4 μg) was used for DNAse treatment and 1 μg pure mRNA was used for cDNA synthesis using an iScript cDNA synthesis kit (Biorad) as per the manufacturer’s instructions. Primers for *Il12a* and *IL12b* were used in conjunction with SSOAdvanced Universal SYBR Green Supermix (Biorad) to quantitate relative levels of these genes in the lung (See S1 Table). qRT-PCR analyses was performed using a Quantstudio 7 Flex Real Time PCR System (Applied Biosystems).

### Cell sorting and quantitation of live *L*. *pneumophila*

Cells were prepared and pooled from whole lungs of *L*. *pneumophila* infected C57BL/6 or IFNγ^-/-^ mice as described. Lung CD11c^+^ cells were enriched via positive selection with an automated magnetic bead separation device using anti-PE microbeads (Miltenyi) against CD11c-PE. Neutrophils were obtained from the negative flow through fraction. Cells were stained for flow sorting using a Beckton Dickinson MoFlo Astrios. Sorted cells were lysed with 0.05% w/v digitonin (Sigma) and lysate plated on selective BCYE agar.

### Adoptive transfer

For adoptive transfer studies, CD8^+^ T cells were isolated from spleens of gBT-I.IFNγ-YFP or gBT-I.IFNγ^*-/-*^ mice. T cells were stimulated *in vitro* with splenocytes loaded with gB peptide as previous [57] for 4–5 days to allow cellular activation and expansion. Briefly, splenocytes were harvested from C57BL/6 mice and incubated in Hank’s Balanced Salt Solution with 0.1 μg/mL gB_498-505_ peptide (GL Biochem) for 45 minutes at 37°C. Splenocytes from gBT-I mice were harvested into RPMI-1640 containing 10% v/v FCS, 100 μM L-glutamine (Astral Scientific), 250 μM HEPES (Sigma Aldrich), 2.5 μM 2-mercaptoethanol (Gibco), 5 U/mL benzylpenicillin (CSL Limited), 0.15 μg/mL LPS (Sigma Aldrich) and 5 mg/mL streptomycin (Sigma Aldrich). gB-pulsed splenocytes were added to cultured gBT-I splenocytes and cells were incubated for 4–5 days at 37°C with 6.5% CO_2_. Cells were split 1:1 on days 2 and 3, and incubated with 25 U/mL recombinant human IL2 (Peprotech). On day 4–5, 1 x 10^7^ gBT-I cells were injected intravenously into recipient mice. Approximately 90–95% of transferred gBT-I T cells expressed Vα2, the Vα of the gBT-I TCR. After 30 days mice were infected with *L*. *pneumophila*.

### Experimental design and statistical analysis

In cases where pooled data was used, each experiment included mice from all experimental groups and data from all mice was used in final analyses.

All comparisons were made using unpaired, two-tailed Mann-Whitney U-test with GraphPad Prism software.

### Ethics statement

All animal experiments were performed with approval from the University of Melbourne Animal Ethics Committee that operates under the *Australian code for the care and use of animals for scientific purposes (2013)*. Ethics IDs were 1312836 and 1112061. Animal facilities operate under licenses from the Bureau of Animal Welfare of the Victorian Government.

## Supporting Information

S1 Fig Inflammatory cells in lungs of CCR2^-/-^ mice after *L*. *pneumophila* infection. A-F. Wild type C57BL/6 or CCR2^-/-^ mice were infected with *L*. *pneumophila* and analysed for the indicated cell types in the lung. Cells identified as described in the main text. Mean ± SEM is shown. A-D, n ≥ 11 for all groups and pooled from ≥ 3 separate experiments. E, F, n ≥ 5 for all groups and pooled from ≥ 2 separate experiments. *. P < 0.05, ****. P < 0.001.
(TIF)

S2 Fig Validation of cytokine reporter transgenic mice. A. Dendritic cells were purified from spleens of IL12p40-YFP mice, placed in culture and left either untreated (Control) or cultured with 0.5 μM of CpG1668 for 16 hours. Cells were then harvested, permeabilised and stained with IL12p40 antibody (C17.8, eBioscience) before analysis by flow cytometry. B. T cells were purified from spleens of IFNγ-YFP mice placed in culture and left either untreated (Control) or cultured with 10 μg/mL of anti-CD3 antibody and 10 μg/mL of anti-CD28 antibody for 16 hours. Cells were then harvested, permeabilised and stained with IFNγ antibody (XMG1.2, eBioscience) before analysis by flow cytometry.
(TIF)

S3 Fig IL18 does not influence *L*. *pneumophila* clearance in the lung. C57BL/6 and IL18^-/-^ mice were infected with *L*. *pneumophila*. A. IFNγ levels in BALF 2 days after infection. B. *L*. *pneumophila* CFU in lungs of indicated mouse strains. Data is pooled from 2–3 independent experiments. A. Each dot represents one mouse. B. Mean ± SEM is shown. n ≥ 6 for each time point. ** *p* < 0.005, NS = not significant.
(TIF)

S1 Table Antibodies, tetramers and primers. List of antibodies, tetramers and primers used in this study.
(PDF)
