# Supplementary figures and images for: Cooperation between Monocyte-Derived Cells and Lymphoid Cells in the Acute Response to a Bacterial Lung Pathogen
Source: PLoS Pathog. 2016 Jun 14;12(6):e1005691. doi: 10.1371/journal.ppat.1005691 (PMC4907431; doi:10.1371/journal.ppat.1005691)

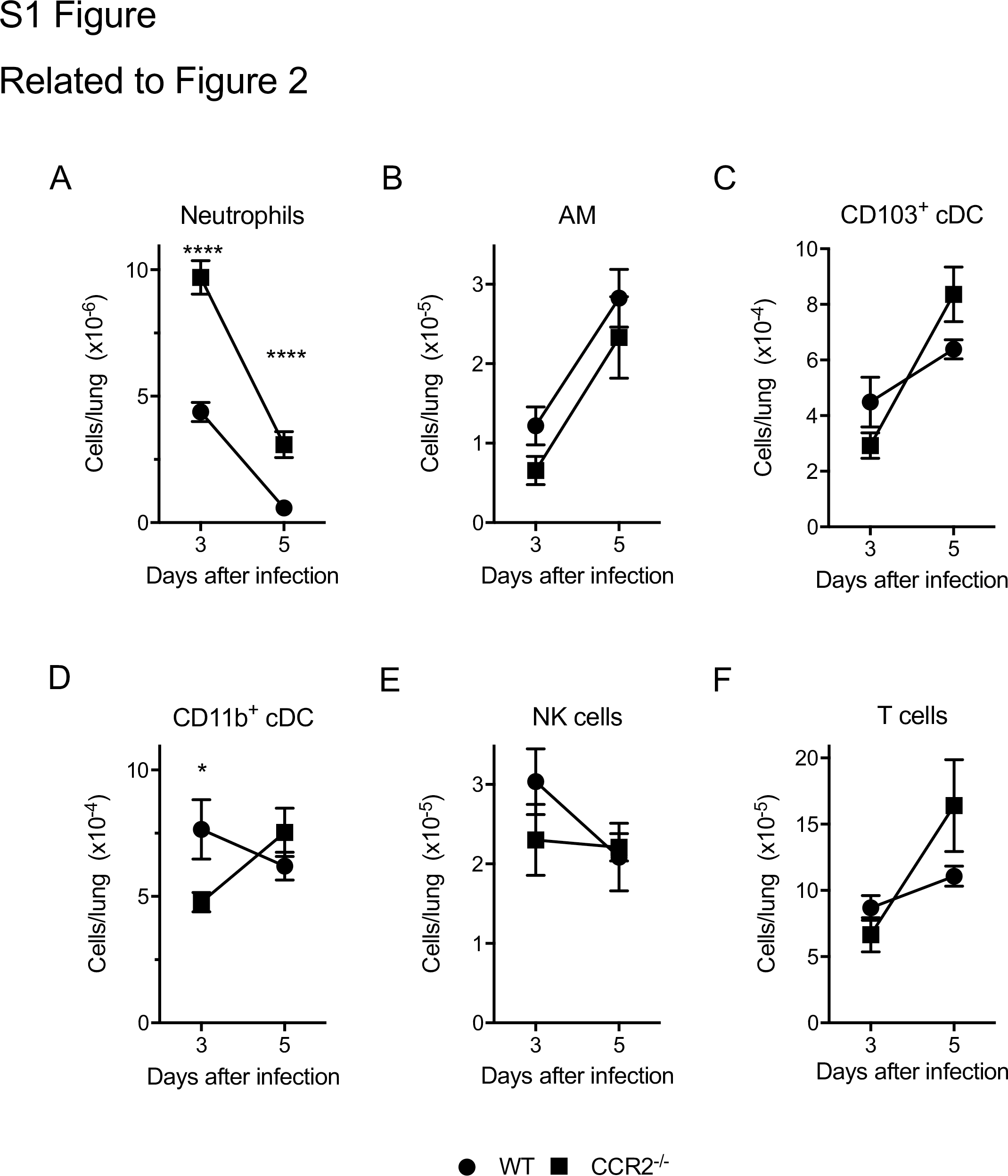

Supplement: S1 Fig — A-F. Wild type C57BL/6 or CCR2-/- mice were infected with L. pneumophila and analysed for the indicated cell types in the lung. Cells identified as described in the main text. Mean ± SEM is shown. A-D, n ≥ 11 for all groups and pooled from ≥ 3 separate experiments. E, F, n ≥ 5 for all groups and pooled from ≥ 2 separate experiments. *. P < 0.05, ****. P < 0.001. (TIF) [file ppat.1005691.s001.tif]

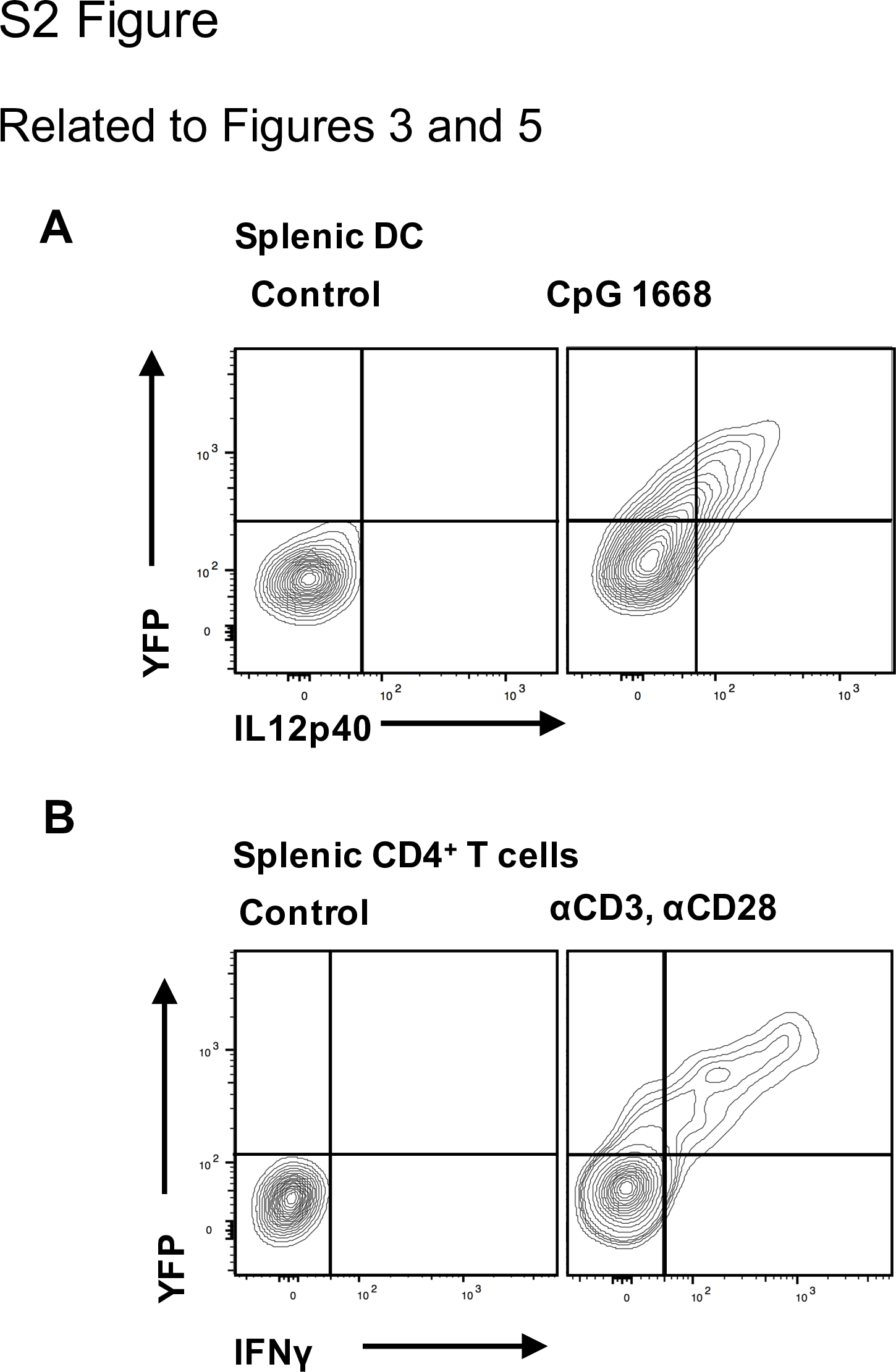

Supplement: S2 Fig — A. Dendritic cells were purified from spleens of IL12p40-YFP mice, placed in culture and left either untreated (Control) or cultured with 0.5 μM of CpG1668 for 16 hours. Cells were then harvested, permeabilised and stained with IL12p40 antibody (C17.8, eBioscience) before analysis by flow cytometry. B. T cells were purified from spleens of IFNγ-YFP mice placed in culture and left either untreated (Control) or cultured with 10 μg/mL of anti-CD3 antibody and 10 μg/mL of anti-CD28 antibody for 16 hours. Cells were then harvested, permeabilised and stained with IFNγ antibody (XMG1.2, eBioscience) before analysis by flow cytometry. (TIF) [file ppat.1005691.s002.tif]

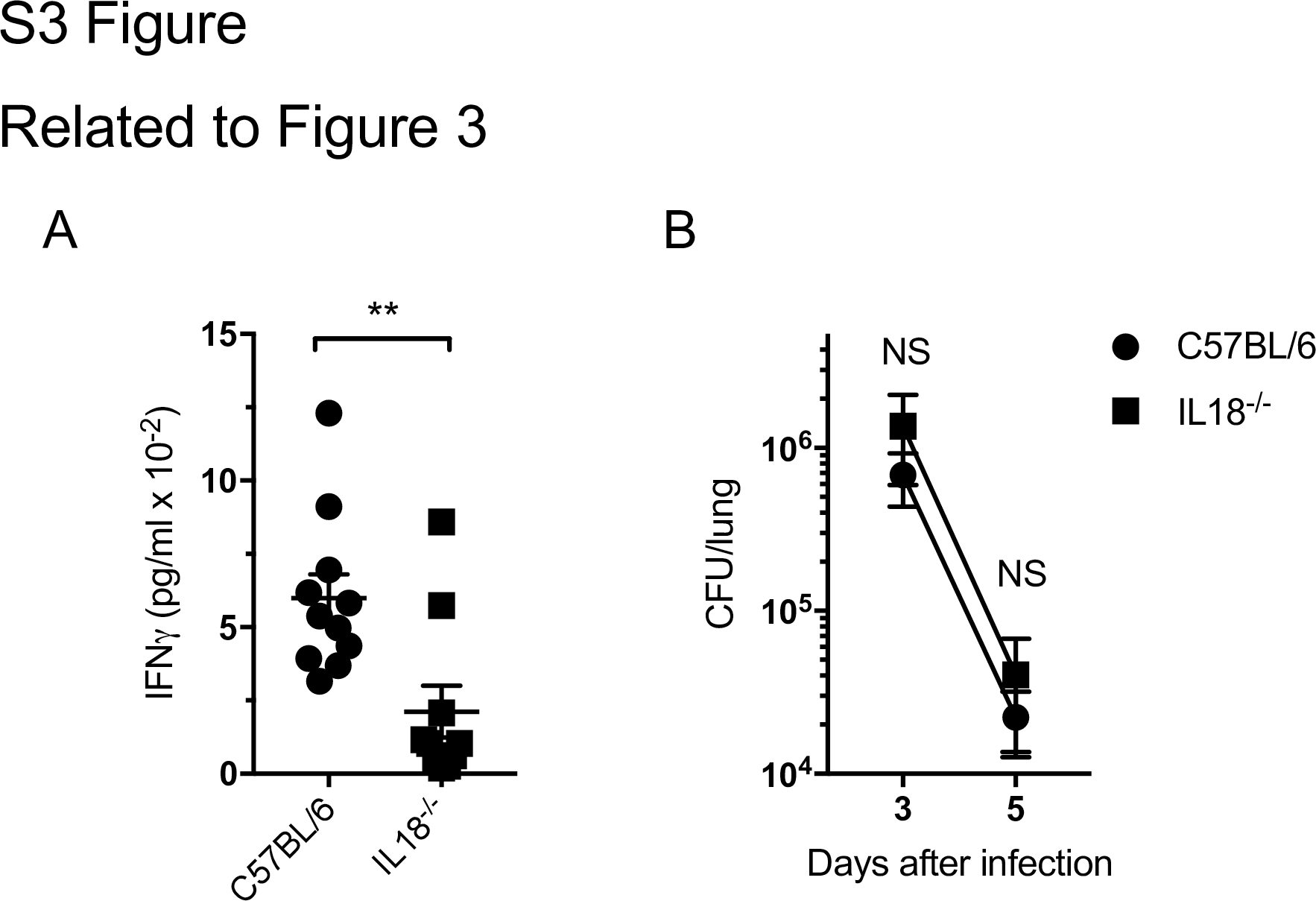

Supplement: S3 Fig — C57BL/6 and IL18-/- mice were infected with L. pneumophila. A. IFNγ levels in BALF 2 days after infection. B. L. pneumophila CFU in lungs of indicated mouse strains. Data is pooled from 2–3 independent experiments. A. Each dot represents one mouse. B. Mean ± SEM is shown. n ≥ 6 for each time point. ** p < 0.005, NS = not significant. (TIF) [file ppat.1005691.s003.tif]
