# Supplementary material for: Cooperation between Monocyte-Derived Cells and Lymphoid Cells in the Acute Response to a Bacterial Lung Pathogen
Source: PLoS Pathog. 2016 Jun 14;12(6):e1005691. doi: 10.1371/journal.ppat.1005691 (PMC4907431; doi:10.1371/journal.ppat.1005691)
Supplement: S1 Table — List of antibodies, tetramers and primers used in this study. (PDF) [file ppat.1005691.s004.pdf]

S1 Table. Antibodies, tetramers and primers

Antibodies

Antigen	Clone	Conjugate	Company	Reference
CD3ε	145-2C11	PerCP-Cy5.5	Biolegend	100328
CD4	RM4-5	eFluor® 450	eBioscience	48-0042
		PE-CF594	BD Horizon	562285
CD8α	53-6.7	APC	eBioscience	17-0081
		PE-Cyanine 7	eBioscience	25-0081
CD11b	M1/70	APC-eFluor® 780	eBioscience	47-0112
CD11c	N418	eFluor® 450	eBioscience	48-0114
		PE	eBioscience	12-0114
	HL3	PE	BD Pharmingen	553802
CD16/32	93	Unconjugated	eBioscience	16-0161
CD44	IM7	Biotinylated	eBioscience	13-0441
CD45	30-F11	PE-Cyanine 7	eBioscience	25-0451
		Biotinylated	eBioscience	13-0451
		V500	BD Horizon	561487
CD45.1	A20	PE	BD Pharmingen	553776
		Biotin	BD Pharmingen	553774
CD45.2	104	FITC	eBioscience	11-0454
		BV421	BD Pharmingen	562895
CD62L	MEL-14	PE-Cyanine 7	eBioscience	25-0621
		FITC	BD Pharmingen	553150
		PE	BD Pharmingen	553151
CD64	X54-5/7.1	Alexa Fluor® 647	BD Pharmingen	558539
CD103	2E7	Biotinylated	eBioscience	13-1031
FcεRI	MAR-1	PE-Cyanine 7	eBioscience	25-5898
I-A/I-E	M5/114.15.2	PerCP-Cy5.5	BD Pharmingen	562363
IFNγ	XMG1.2	APC	eBioscience	17-7311
IL12p40	C17.8	Biotinylated	eBioscience	13-7123
Legionella	Polyclonal	FITC	ViroStat	6053
Ly6C	AL21	Biotinylated	BD Pharmingen	557359
Ly6G	1A8	PerCP-Cy5.5	BD Pharmingen	560602
		FITC	BD Pharmingen	551460
NK1.1	PK136	APC	BD Pharmingen	550627
NKp46	29A1.4	Biotinylated	eBioscience	13-3351
Rabbit IgG	N/A	FITC	eBioscience	11-4614
Siglec F	E50-2440	PE	BD Pharmingen	552126
		BV421	BD Horizon	562681
TCRβ	H57-597	APC-eFluor® 780	eBioscience	47-5961
		PE	BD Pharmingen	553172
TCRγδ	GL3	PE	BD Pharmingen	553178

Tetramers

CD1d-tetramer	PE	From Prof Dale Godfrey,Uni Melbourne
MR1-tetramer	BV421	From Prof James McCluskey, Uni Melboune

Secondary Staining Reagents

Streptavidin	PE	eBioscience	12-4317
	APC	BD Pharmingen	554067
	PE-CF594	BD Horizon	562284
	V500	BD Horizon	561419

Primer Sequences

Il12a

Forward: 5’-CCG AAA CCT GCT GAA GAC CA-3’
Reverse: 5’-GGT TTG GTC CCG TGT GAT GT-3’

Il12b

Forward: 5’-GAC CAT CAC TGT CAA AGA GTT TC-3’
Reverse: 5’-AGG AAA GTC TTG TTT TTG AAA-3’
